# Supplementary material for: Cell Cycle Dynamics in the Microalga Tisochrysis lutea: Influence of Light Duration and Drugs
Source: Cells. 2024 Nov 20;13(22):1925. doi: 10.3390/cells13221925 (PMC11592524; doi:10.3390/cells13221925)
Supplement: Supplementary file 1 [file cells-13-01925-s001.zip › cells-3298421-supplementary.pdf]

Supporting Figures

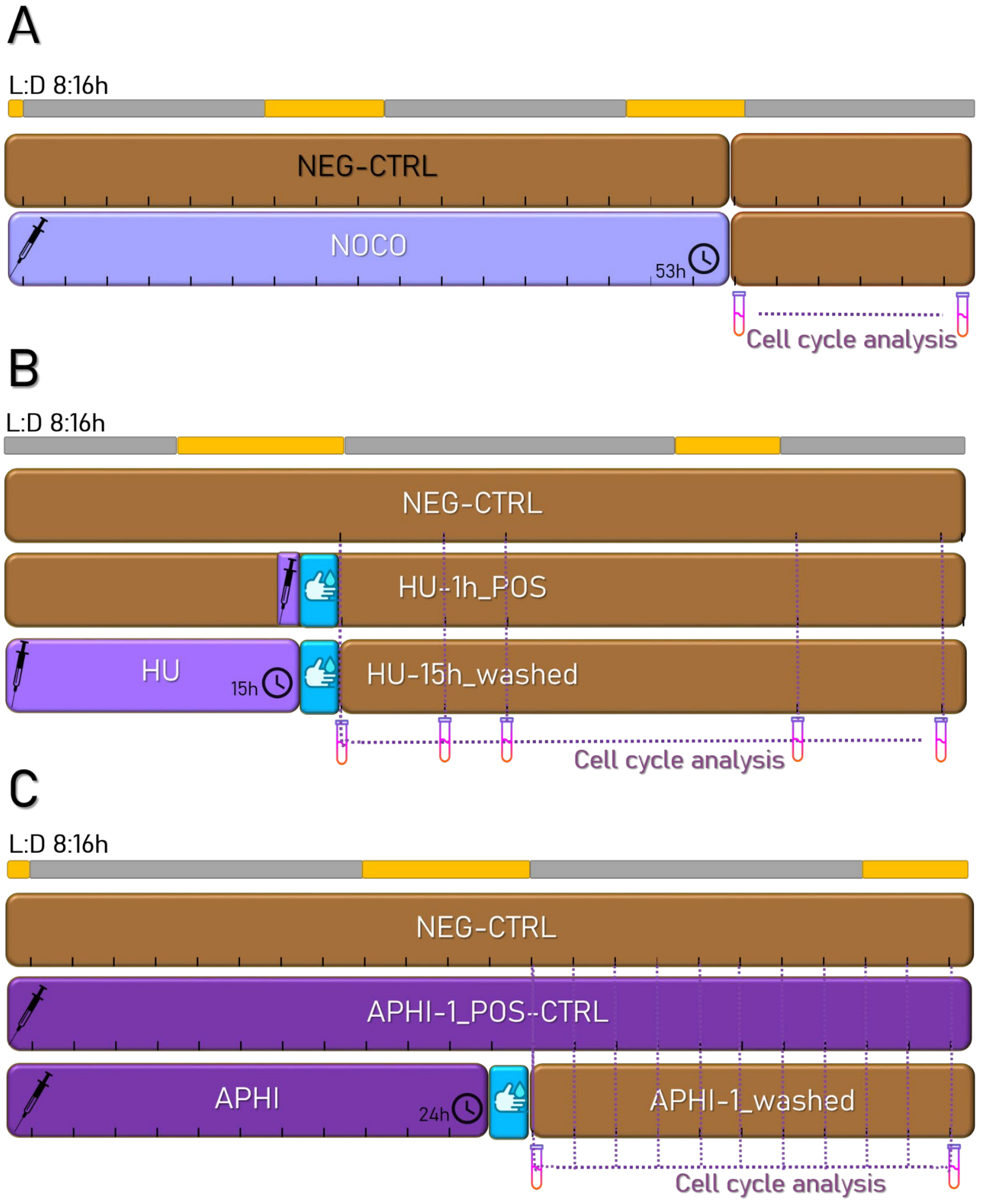

Supplementary Figure S1: Sequence of events for chemical synchronization of *T. lutea* cultures. Cells were treated with A: nocodazole (NOCO, Cf = 10 μg mL^{-1}) during 53-h incubation and were then harvested every two hours during 8 h monitoring with flow cytometry (without washing). B: hydroxyurea (Cf = 0.64 μg mL^{-1}) either after 1 h incubation (HU-1h_POS) or after 15 h incubation (HU-15h_washed). Following incubation, treated cells were washed of HU and sampled at three

points in the cycle (L7h, D6h, and D9h) for two consecutive days. C: aphidicolin (Cf = 1 μg mL^{-1}) during the entire experiment (APHI-1_POS-CTRL) or after a 24-h incubation (APHI-1_washed) followed by drug rinse. A control with no exposure to the blocking agent (NEG-CTRL) was simultaneously performed for each experiment.

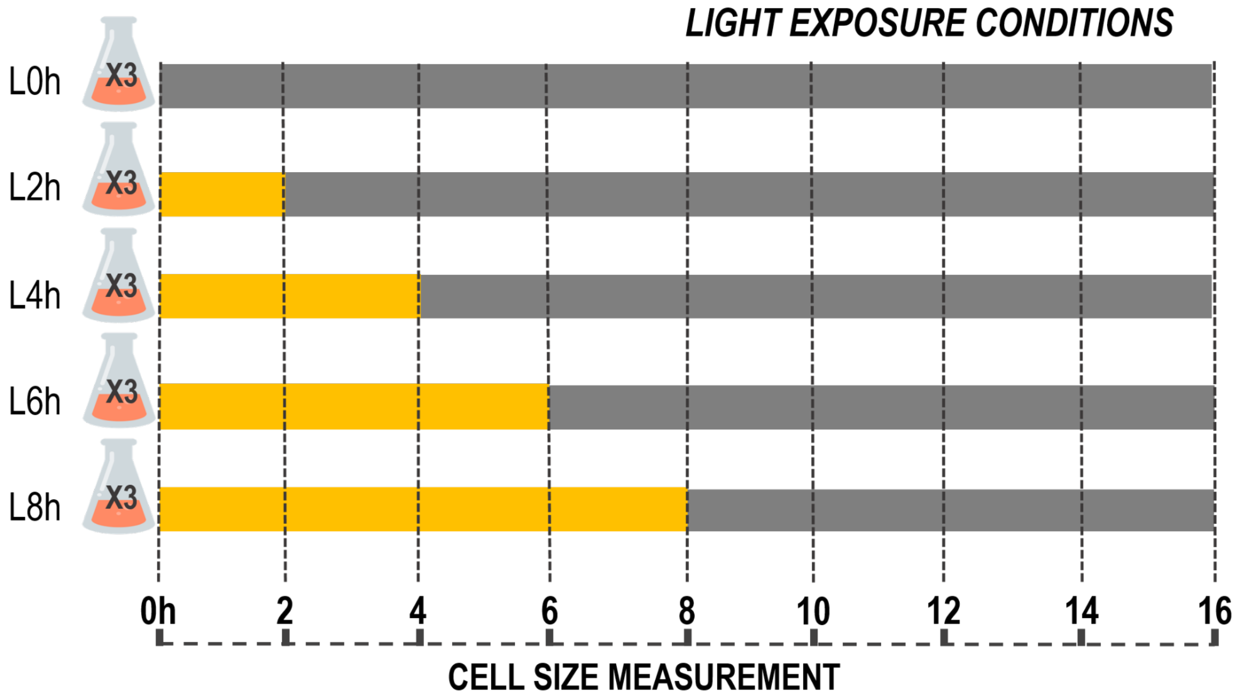

Supplementary Figure S2: Experimental setup involved growth-interruption trials on *T. lutea* cultures, initially exposed to 16 hours of darkness, followed by monitoring for 16 hours under varying durations of light exposure (0 h, 2 h, 3 h, 4 h, 6 h, 8 h) at an intensity of 140 µmol photons m^{-2} s^{-1} (Mean ± SE, N = 3). Following the light exposure, the cultures were placed in darkness to halt photosynthetic growth. The diagrams illustrate the specific photoperiod applied to each experimental condition.

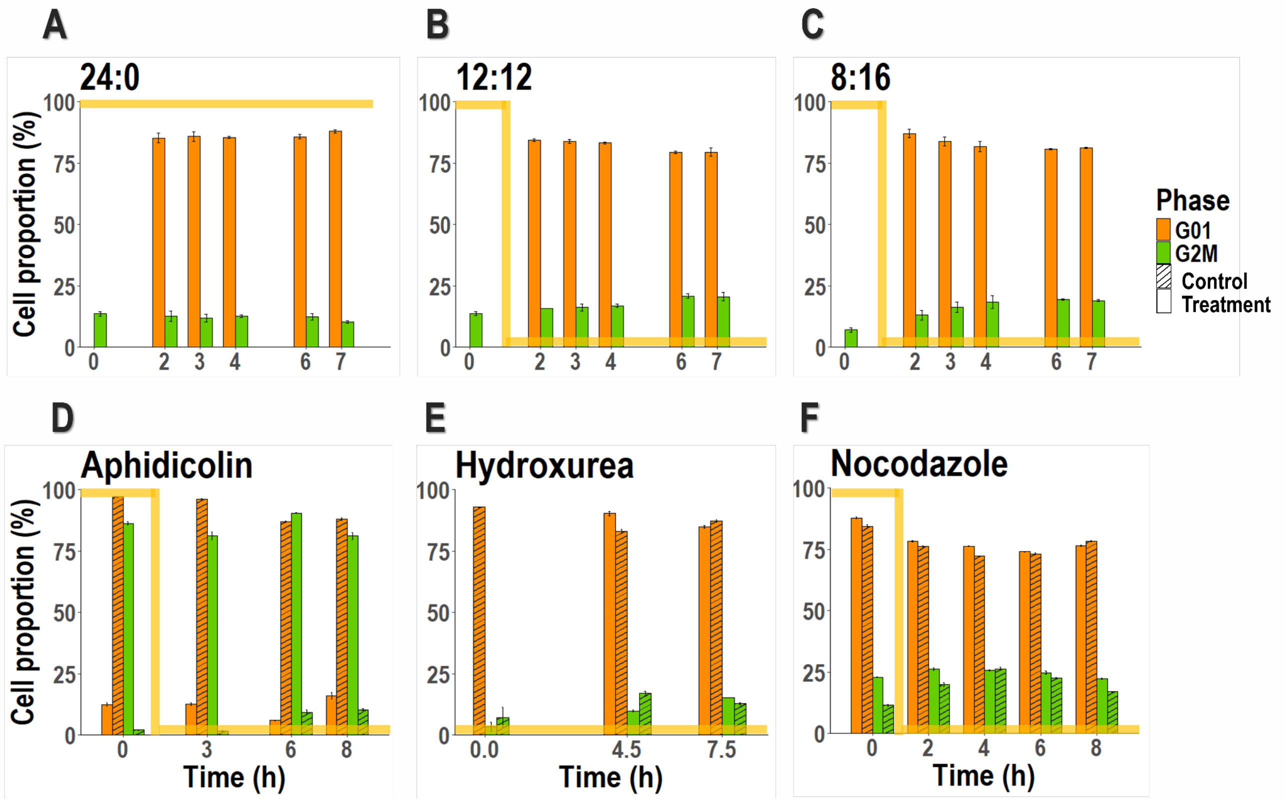

Supplementary Figure S3: Changes in the proportions (%) of Tisochrysis lutea cells in the G0/1 and G2/M phases of the cell cycle, identified via flow cytometry, under different light-dark (L:D) regimes: 24:0 (A), 12:12 (B), and 8:16 (C). Additional conditions include treatments with chemical agents: aphidicolin (final concentration = 1 µg mL^{-1} after 24-hour incubation), hydroxyurea (final concentration = 0.64 µg mL^{-1} after 15-hour incubation), and nocodazole (final concentration = 10 µg mL^{-1} after 53-hour incubation). Data are presented as means ± standard error (N = 3) throughout the experiment. The yellow line indicates light intensity, where a value of 100 represents daylight (140 µmol photons m^{-2} s^{-1}) and 0 represents complete darkness. It is important to note that comparisons between the trends observed should be avoided due to differences in sampling designs across experiments.

Supplementary Table S1: Raw data depicting volume distributions (μm^3) of *T. lutea* triplicate cultures (R1, R2, R3) under an 8-hour daytime photoperiod at the end of the dark period (T16h: Post-division) and at the end of the light period (T8h: Pre-division). The Δ D/L is the calculated difference between Post-division (new daughter cells formed) and Pre-division (mother cells ready to divide) cell numbers. The inversion point (from positive to negative values) enables the estimation of the minimum cell volume or 'sizer' (depicted by a dotted line) at the commitment point (CP), for each replicate.

Cellular volume (µm3)	Δ D/L			Post-division cells (cell counts)			Pre-division cells (cell counts)		
					Dark			Light	
	R1	*R2*	*R3*	*R1*	*R2*	*R3*	*R1*	*R2*	*R3*
21,43	9	11	2	15	18	12	6	7	10
22,18	14	29	23	26	34	31	12	5	8
22,94	36	22	10	42	35	26	6	13	16
23,74	47	46	48	55	58	59	8	12	11
24,56	74	71	62	84	85	71	10	14	9
25,41	100	78	96	107	94	104	7	16	8
26,29	129	135	156	139	149	161	10	14	5
27,20	196	189	188	206	201	198	10	12	10
28,14	244	238	251	259	250	259	15	12	8
29,11	288	286	337	301	297	346	13	11	9
30,12	326	347	368	340	373	380	14	26	12
31,16	395	378	436	414	414	458	19	36	22
32,24	432	398	444	468	432	473	36	34	29
33,35	385	452	434	427	484	483	42	32	49
34,51	444	380	406	492	453	472	48	73	66
35,70	400	440	389	485	522	453	85	82	64
36,94	443	374	414	541	483	524	98	109	110
38,21	389	394	376	532	550	514	143	156	138
39,54	350	352	290	520	521	494	170	169	204
40,90	310	319	320	536	534	554	226	215	234
42,32	275	292	311	546	539	535	271	247	224
43,78	219	201	232	518	517	531	299	316	299
45,30	198	173	173	546	506	542	348	333	369
46,86	178	200	139	545	566	526	367	366	387
48,49	120	107	137	544	527	549	424	420	412
50,16	119	94	111	540	547	551	421	453	440
51,90	48	49	70	510	496	539	462	447	469
53,69	22	24	120	494	534	567	472	510	447
**55,55**	-51	15	13	481	499	486	532	484	473
**57,47**	-30	-54	-56	447	459	436	477	513	492
59,46	-89	-156	-126	378	383	394	467	539	520
61,52	-150	-142	-186	369	313	352	519	455	538
63,65	-194	-205	-156	276	298	315	470	503	471
65,85	-216	-241	-225	257	240	243	473	481	468
68,13	-249	-278	-264	172	194	208	421	472	472
70,49	-264	-283	-277	160	141	160	424	424	437
72,92	-333	-253	-251	124	157	141	457	410	392
75,45	-248	-277	-222	105	107	122	353	384	344
78,06	-226	-245	-226	112	108	95	338	353	321
80,76	-237	-184	-195	83	85	97	320	269	292
83,55	-186	-222	-178	68	50	68	254	272	246
86,44	-173	-157	-154	55	51	60	228	208	214
89,43	-160	-97	-135	49	52	44	209	149	179
92,53	-124	-112	-130	29	36	23	153	148	153
95,73	-84	-85	-86	25	27	33	109	112	119
99,04	-71	-63	-85	18	26	19	89	89	104
102,47	-48	-64	-46	15	16	21	63	80	67
106,01	-44	-48	-32	11	11	18	55	59	50
109,68	-33	-33	-21	17	15	13	50	48	34
113,47	-20	-17	-22	12	9	6	32	26	28
117,40	-16	-20	-16	8	11	16	24	31	32
121,46	-6	-19	-8	10	5	5	16	24	13
125,66	-5	-2	-1	5	8	8	10	10	9
130,01	-4	-2	-8	6	6	1	10	8	9
134,51	-5	-4	-2	2	3	5	7	7	7
139,16	-1	-1	1	5	6	3	6	7	2
